# Proposed New Rule for C.M.B. Candidates

**Published:** 1920-06-05

**Authors:** 


*To the Editor of* The Hospital.

### Proposed New Rule for C.M.B. Candidates.

*To the Editor of* The Hospital.

Sir,—Will you kindly let me draw the attention of your readers to the fact that, at the last meeting of the Central Midwives' Board, the Board—owing to so many nurses having the C.M.B. certificate and not practising—"has been led to consider whether it might be wise to attach as a condition to the award of the C.M.B. certificate that the holder must agree to practise for a given period"? For this to apply to trained nurses who have already spent three or four years at least in training would be very hard and unfair. The C.M.B. certificate is now so often necessary as a finish to a nurse's general training as a help to better posts, public health work, and work abroad. I trust that all who have the welfare of trained nurses at heart will see to this.—I am, yours truly,

"Fair Play."

